# GalaxyTBM: template-based modeling by building a reliable core and refining unreliable local regions

**DOI:** 10.1186/1471-2105-13-198

**Published:** 2012-08-10

**Authors:** Junsu Ko, Hahnbeom Park, Chaok Seok

**Affiliations:** 1 Department of Chemistry, Seoul National University, Seoul, 151-747, Republic of Korea

**Keywords:** Protein structure prediction, Model refinement, Loop modeling, Terminus modeling

## Abstract

**Background:**

Protein structures can be reliably predicted by template-based modeling (TBM) when experimental structures of homologous proteins are available. However, it is challenging to obtain structures more accurate than the single best templates by either combining information from multiple templates or by modeling regions that vary among templates or are not covered by any templates.

**Results:**

We introduce GalaxyTBM, a new TBM method in which the more reliable core region is modeled first from multiple templates and less reliable, variable local regions, such as loops or termini, are then detected and re-modeled by an *ab initio* method. This TBM method is based on “Seok-server,” which was tested in CASP9 and assessed to be amongst the top TBM servers. The accuracy of the initial core modeling is enhanced by focusing on more conserved regions in the multiple-template selection and multiple sequence alignment stages. Additional improvement is achieved by *ab initio* modeling of up to 3 unreliable local regions in the fixed framework of the core structure. Overall, GalaxyTBM reproduced the performance of Seok-server, with GalaxyTBM and Seok-server resulting in average GDT-TS of 68.1 and 68.4, respectively, when tested on 68 single-domain CASP9 TBM targets. For application to multi-domain proteins, GalaxyTBM must be combined with domain-splitting methods.

**Conclusion:**

Application of GalaxyTBM to CASP9 targets demonstrates that accurate protein structure prediction is possible by use of a multiple-template-based approach, and *ab initio* modeling of variable regions can further enhance the model quality.

## Background

Three-dimensional protein structures provide invaluable insights into the molecular basis of protein functions, and such insights are essential for rational design of molecules regulating these functions. Nowadays, in an increasing number of cases, it has become possible to model protein structures with acceptable accuracy by employing much less effort than that required in experimental methods. Progress in computational protein structure prediction has been boosted by methodological improvements in the technique called template-based modeling (TBM), which uses experimental structures of homologous proteins as templates. As biological sequence and structure databases expand continuously, TBM is expected to become an even more promising tool for practical molecular biology, pharmaceutical chemistry, and protein engineering problems [1].

Template-based modeling, also called homology modeling or comparative modeling, generally consists of the following steps [1,2]: (1) identification of homologous proteins with known structures to be used as templates; (2) alignment of the sequences of the target and templates; (3) creation of model structures from the alignment; and (4) refinement of the models. Contemporary methods usually treat each stage separately, and the full TBM procedure can therefore be established by combining methods for each of the above stages.

Despite recent progresses, there still remain challenges for each stage mentioned above. One of the important challenges is how to optimally combine information from multiple templates to build a single model when experimental structures of multiple homologues are available. Using multiple templates rather than a single template offers several obvious benefits: the possibility of including a better template increases, and the fraction of the target sequence covered by templates is extended [3-5]. In addition, different regions in template structures may be combined to produce a more accurate model [3]. However, in practice, it is complicated to combine information from multiple templates in an optimal way [6]. Since the average quality of multiple templates is bound to be worse than that of the single best template, using multiple templates is associated with a rather large risk of contaminating reliable information from the best template. To overcome this problem, various approaches have been proposed [1,7,8]. Most of them heavily rely on a single top template while additional templates are used to fill the gaps not covered by the top template [3,9].

Another challenge is to model structurally variable regions among templates or those regions not covered by any templates, which we call ULRs (unreliable local regions). Unless the target sequence is quite similar to those of the templates (for example, with sequence identity > 30%), the expected quality of template-based models could be limited by such regions. Moreover, such ill-conserved regions where sequence insertion/deletion occurs may not be the subject of typical TBM. Despite previous efforts, progress in modeling such regions seems to be rather limited [10]. Since high-resolution models are required for practical applications, it is doubtless that better ULR modeling is essential.

We recognize that the above 2 challenges are not independent of each other. For example, the performance of ULR modeling can be limited by the quality of the framework structure constructed from multiple templates [10,11]. We therefore propose a strategy by which both initial TBM and subsequent ULR modeling can benefit from each other. In the initial TBM, we focus on accurate modeling of more conserved regions among multiple templates, without the need to consider potentially unreliable regions since such regions are taken care of in the ULR modeling stage. In the ULR modeling stage, we fix the more reliable core structure so as not to deteriorate the overall model quality by potentially less reliable *ab initio* ULR modeling. Therefore, ULRs can be modeled in a more accurate framework structure, and the conformational search space for ULR modeling is also effectively reduced to the local regions. Related approaches that construct a reliable core and refine unreliable regions have been proposed previously [12,13]. The difference between our approach and these is that we put more stress on the “accuracy” (rather than the “coverage”) of the core structure in the initial TBM stage (See METHODS for details).

We call this new method GalaxyTBM, as it is based on the GALAXY molecular modeling package [11,14-16]. GalaxyTBM employs a multiple-template method designed to produce reliable core structures by rescoring HHsearch [17] results for multiple-template selection and by core sequence alignment using PROMALS3D [18]. Model building from the alignment and subsequent ULR modeling is performed using optimization modules in GALAXY [11,16].

All components of the prediction pipeline were tested in the 9th critical assessment of techniques for protein structure prediction (CASP9) as a predictor named “Seok-server.” According to the official results of CASP9, Seok-server is recognized as one of the top 6 servers [19]. Since the prediction strategy for Seok-server had to be modified a few times during CASP9, as the method was immature at the beginning, the most recent version, GalaxyTBM, is presented here. When GalaxyTBM was tested on 68 single-domain CASP9 TBM targets, fixing the structure database at the version with which Seok-server was used during CASP9, it reproduced the performance of Seok-server (average GDT-TS of 68.1, compared to 68.4 for Seok-server). Performance of the TBM pipeline was evaluated by analyzing the improvements achieved at each stage. Merits of the new components in the pipeline over other TBM methods are also discussed.

## Results and discussion

### Rescoring of HHsearch results improves the template quality

We used a simple but effective rescoring strategy to select multiple templates from the homologues detected by HHsearch [17], as described in the METHODS section. Here, we analyzed the performance of the rescoring method in terms of the quality of the top ranker compared to that of the HHsearch top ranker. Template quality was measured by a similarity score called TM-score calculated using the TM-align tool [20], which ranges from 0 (no similarity) to 1 (same as the native structure). Improvement achieved by the selection scheme of “multiple” templates is discussed in the next subsection.

Overall, top rankers obtained by the rescoring scheme were closer to the native structures of the target proteins than the HHsearch top rankers, when tested on the 68 single-domain CASP9 TBM targets. Different proteins ranked as top by rescoring in 19 out of the 68 cases, with an average improvement of 0.046 in TM-score. TM-score increased for 15 out of the 19 targets and decreased for the remaining 4 targets, with average increases of 0.072 and −0.033, respectively. A paired t-test for the TM-score changes for the 19 targets showed that the improvement is statistically significant, with a P-value of 0.0072.

**Figure 1:**
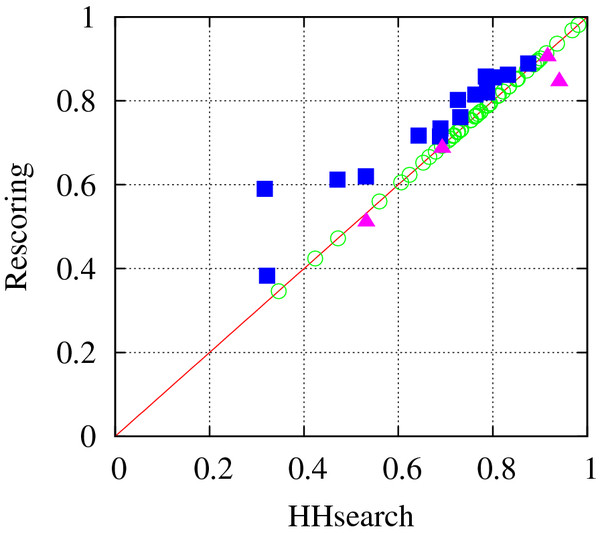
**Comparison of the template quality (measured by TM-score) of the top ranker assessed by HHsearch and by rescoring.** The rescoring strategy placed better templates as top in 14 cases (squares) and worse templates as top in 5 cases (triangles) out of the 68 test cases.

As can be seen from Figure 1, the improvement did not strongly correlate with target difficulty. It is also notable that outstanding improvement was obtained in some cases. For example, the top ranker in the HHsearch for T0564 had a low TM-score of 0.317, but the rescoring scheme ranked a protein with a TM-score of 0.590 as the first, although this protein ranked very low (2524th) in the original HHsearch. These improvements could be primarily attributed to the greater consideration of the secondary structure score when scoring more difficult targets (i.e., targets with more distant homologues). In other words, when sequence conservation between target protein and homologues is low, information on secondary structure conservation can be more helpful in selecting closer homologues.

### Multiple-template information improves the model quality

**Figure 2:**
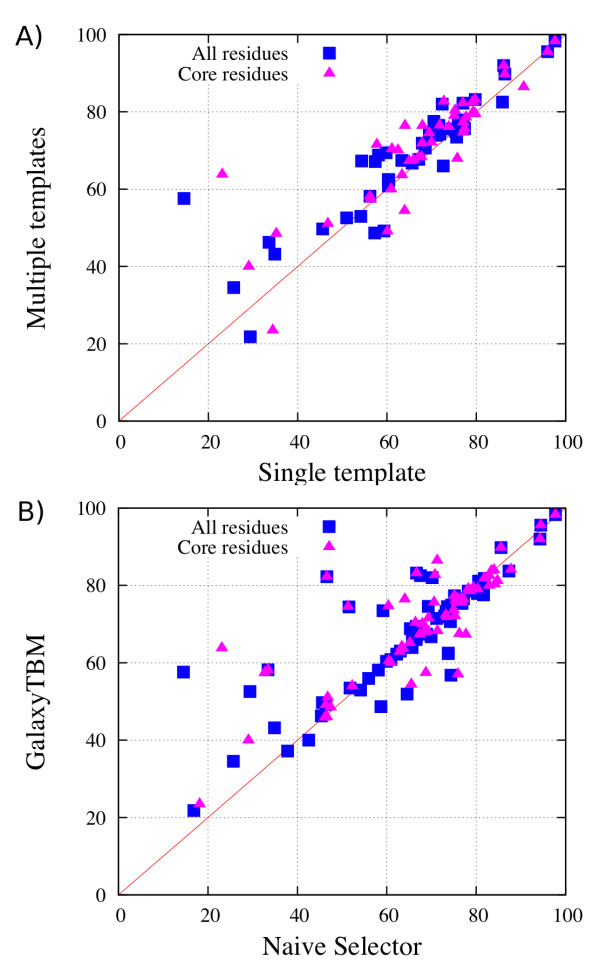
Quality of models (measured by average GDT-TS scores of 100 models) obtained from the multiple-template approach compared to those from A) a single-template approach and B) a naïve multiple template selection method for the whole domain (squares) and only for the core residues (triangles) aligned to the top-ranking template.

In addition to selecting top rankers successfully, the template rescoring method is also effective in providing candidates for multiple templates. Multiple templates were selected by filtering out structural outliers from the candidates after core structure alignment (see METHODS for detail). To assess the improvement achieved by using multiple templates, model structures built from the multiple templates were compared with those built from the single top template (after rescoring) (Figure 2A) and those from a naïve multiple template selection method that considers all proteins with HHsearch e-values < 10^-10^ (Figure 2B). The average number of templates selected by GalaxyTBM and the naïve method was 3.04 and 8.85, respectively. For each target and for each method, 100 models were generated by MODELLER [21], and GDT-TS score (a measure of similarity to the native structure [22,23], ranging from 0 for no similarity to 100 for the native structure) averaged over them is reported. Although TM-score is used for measuring the quality of a “template” which has a different sequence from the native protein, the GDT-TS measure is used for assessing the quality of a “model” with the same sequence.

By using the current multiple template approach, GDT-TS improved when compared to the values obtained using the single-template approach (the sum over 68 domains increased by 3.4% from 4429.3 to 4580.9 with an average improvement of 2.23 per domain) and the naïve multiple-template method (the sum increased by 3.8% from 4412.7 to 4580.9 with an average improvement of 2.47 per domain). The GDT-TS improvement is statistically significant, with P-values of 0.006 and 0.02, respectively. Improvement over the naïve method was prominent when high-ranking proteins by HHsearch have diverse structures, implying that the current multiple-template selection scheme that excludes dissimilar structures is fairly successful. For example, for T0539, mean GDT-TS of generated models is 75.59 and 59.14 by the current approach and by the naïve approach, respectively. Similar type of large GDT-TS improvement of > 5 was also found in T0532, T0552, T0559, T0614, and T0643.

To determine whether the model improvement by the multiple-template approach is a consequence of covering more residues by additional templates, we checked whether a similar improvement was found for core regions (Figure 2). The core region is defined here as the target residues aligned with the single top template by PROMALS3D [18]. The core region covers 31% to 100% of the whole protein, with an average coverage of 85%. As shown in Figure 2A, GDT-TS of the core region was also improved by the current multiple-template method compared to the single-template method. Average GDT-TS improvement was 2.08%, with a P-value of 0.0106.

In conclusion, the current multiple template selection method contributes to improving the core structure by utilizing useful information from additional templates selected by the current pipeline.

### Better optimization during model-building further improves the model quality

In GalaxyTBM, model building is performed by the MODELLER-CSA [24] module implemented in GALAXY. It was previously reported that more thorough optimization of the target restraint function derived by MODELLER is possible with the method, generating model structures more consistent with the restraints [24]. To evaluate the performance of model building in the current pipeline, we compared the structures generated in this stage with the model structures generated simply by using MODELLER [21]. The 2 methods, MODELLER and MODELLER-CSA, use the same sequence alignments, template lists, and therefore the same spatial restraints, and differ only in the optimization method.

As in the previous subsection, 100 model structures were generated for each target. Overall, model building by MODELLER-CSA improved the sum of GDT-TS by 0.6% (from 4580.9 to 4622.2) compared to MODELLER, with a P-value of 0.002. Average GDT-TS improvement was 0.13 for the 25 targets for which single templates were used and 0.87 for the 43 targets for which multiple templates were used. The better GDT-TS improvement in the multiple-template cases can be explained by the fact that the more complex target restraint functions for the multiple-template problems can be better optimized with the more rigorous optimization method [24].

In addition to the backbone structure quality, the side-chain structure quality was also improved by the better optimization during model building. The χ1 accuracy (percentage of the cases in which χ1 is within 30° from the native value) was improved in 65 out of 68 targets, with an average improvement of 5.9%. The χ1 + χ2 accuracy (percentage of the cases in which both χ1 and χ2 are within 30° from the native values) was also improved (improved in 63 out of 68 cases, with an average improvement of 4.5%). This improvement is consistent with the findings of the previous report by Joo *et al.*[24].

### Positive effects of the overall multiple-template strategy

**Figure 3:**
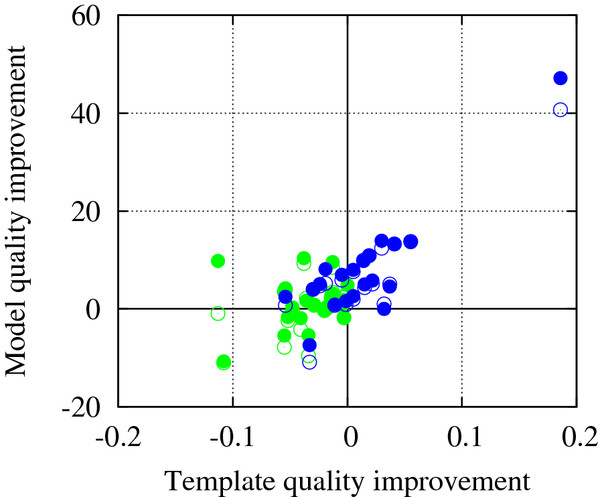
**Model quality improvement achieved by the use of multiple templates (relative to the single-template method, measured by GDT-TS) against average template quality improvement (relative to that of top ranker, measured by TM-score) for the 43 targets for which multiple templates are used.** Only core residues were considered in this comparison. Overall, as the average quality of multiple templates decreased, model quality improvement achieved by the use of multiple templates tended to decrease for both model-building methods, MODELLER (empty dots) and MODELLER-CSA (filled dots). Targets for which the top ranking template was the best-quality template are shown in green and others in blue.

To illustrate the effects of the overall multiple-template strategy in more detail, the relationship between the model quality improvement and the template quality improvement achieved by the use of multiple templates is demonstrated in Figure 3. In this figure, model qualities were measured for the core region, instead of the whole structure, to clarify the impact of using multiple templates. The points in the upper right corner of Figure 3 represent the cases in which templates of higher quality than the top ranker contribute to improving the model quality. This is one of the expected positive effects of using multiple templates. It is particularly intriguing that in a non-negligible number of cases (23 out of 43), model quality was improved even when the average template quality decreased from that of the top single template (upper left corner of the figure), particularly in those where no additional templates had better quality than the top ranker (green dots in the upper left corner). This effect may be attributed in part to the success of the multiple-template selection method, which can pick out additional complementary templates without introducing large negative effects of contaminating good information from the top ranker. Improvement by more thorough optimization in the model-building stage (filled dots compared to open dots) is more pronounced in the left side of the figure, where the average template quality was worse than that of the top ranker.

The above analysis indicates that the positive effects of using multiple templates can be maximized by the current template selection strategy that considers core structure consensus and the more rigorous optimization during model-building, and the common adverse effects caused by including inconsistent templates in typical multiple-template methods can be minimized by use of such a combination.

### ULR refinement also contributes to improvement of the model quality

Here we present the results of the final stage of the pipeline, i.e., refinement of ULRs. A total of 204 ULRs (56 termini and 148 loops) were detected in the initial stage, and 132 ULRs were finally subject to reconstruction following the selection rule described in METHODS. These reconstructed ULRs consisted not only of the regions that were not aligned to any template residues but also of the regions that were structurally inconsistent among templates.

**Figure 4:**
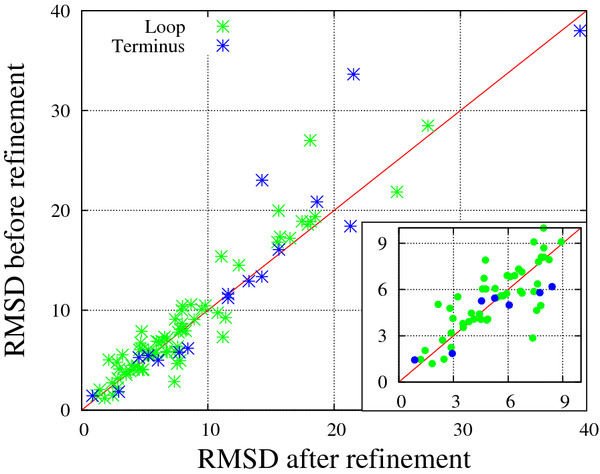
**Comparison of ULR RMSDs (in Å) from the native structure before and after ULR refinement.** RMSD for each ULR was calculated after superimposing the whole structures (native and model), not just the ULRs. Results for ULRs with RMSD < 10 Å are magnified in the inset.

Of the 132 reconstructed ULRs, 45 ULRs corresponded to the regions in which more than half of the residues are disordered in experimental structures and thus were neglected in the following analysis. In Figure 4, RMSDs of the ULR structures (distances from the native structures after superimposing the whole structures, not just the ULR regions) before and after refinement are compared for each ULR. The average improvement in RMSD achieved by refinement was 0.80 Å. The RMSD improvement was statistically significant, with a P-value of 0.0015. Since the current refinement changes only local regions, improvement in the overall structure measured by GDT-TS was rather small. The sum of GDT-TS over 52 domains containing refined ULRs increased by 0.47% (from 3392.8 to 3408.7), with an average increase of 0.31 per domain. Although the improvement was small, it is statistically significant, with a P-value of 0.0083. More accurate prediction of ULR regions is invaluable for functional or design studies that involve such protein-specific local regions. It is also notable that the current refinement result is comparable to the results obtained by Seok-server in CASP9 [19], even though a lighter ULR optimization strategy has been employed.

### Computational cost

Template selection and multiple sequence alignment take a few minutes on a single core. The median time required for model building with MODELLER-CSA and refinement was 6.2 and 1.1 hours, respectively, when 32 cores were used in parallel.

## Conclusions

In this article, we report a new TBM method—GalaxyTBM—that builds reliable core structure from multiple templates and reconstructs unreliable regions by *ab initio* loop or terminus modeling in the fixed framework of the core structure. The current multiple-template strategy maximizes the positive effects of using multiple templates by selecting complementary multiple templates that do not contaminate the information from the best template significantly and by thorough optimization of possibly conflicting template restraints during model building. When model refinement by detection and re-building of unreliable loop or terminus regions is applied, additional improvement in model quality is observed. Several sound elements of the current strategy, such as template rescoring, multiple-template selection based on core-structure alignment, and multiple sequence alignment of core sequences could be easily incorporated into other TBM methods to enhance their performance.

## Methods

**Figure 5:**
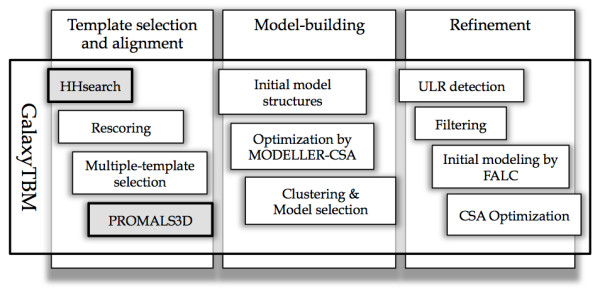
**Schematic flowchart of the overall Galaxy TBM process, which flows from left to right.** Major stages are indicated at the top, and the components of each stage are listed below.

The overall modeling procedure by the GalaxyTBM pipeline is shown in Figure 5, and each stage is explained below.

### Multiple-template selection

Multiple templates were selected by rescoring the results of HHsearch (version 1.5.0) [17], one of the best homologue detection methods available. The protein structure database pdb70, with maximum mutual sequence identity of 70%, was used. The proteins ranked by HHsearch run in the local alignment mode with the Viterbi algorithm were first re-ranked by the rescoring function *S* expressed as a weighted sum of the Z-score of the sequence similarity score, *Z*_seq_, and that of the secondary structure similarity score, *Z*_ss_

(1) $$S=Z_{\text{seq}}+wZ_{\text{ss}}$$

where the raw sequence and secondary structure similarity scores were taken from HHsearch results. The weight factor *w* depends on the target difficulty estimated by the HHsearch probability for the top ranker, *p*, as

(2) $$w=\{\begin{array}{c} 1.0\left(p\geq 90\right)\\ 1.5\left(80\leq p<90\right)\\ 2.0\left(60\leq p<80\right)\\ 2.6\left(p<60\right) \end{array}$$

The probability bins and the corresponding weight factors for rescoring were determined by maximizing the qualities of the top templates for the CASP8 TBM targets using a grid search in pre-set ranges of the parameters.

Multiple templates were then selected from the 20 top-ranked proteins as follows. First, the top 20 proteins were divided into high-rankers (those with the score *S* within 95% of the top ranker’s score) and low-rankers (the remaining ones). Second, those proteins that had dissimilar structures from a “background pool” of structures were removed. The background pool consisted of either the high-rankers or top 3 rankers, whichever was the greatest. The similarity of a protein structure to the background pool was measured by the mean TM-score from the pool structures, and the proteins that had lower similarity than the cut-off value, $m_{\text{pool}}-\alpha \sigma_{\text{pool}}$, were removed, where *m*_pool_ and $\sigma_{\text{pool}}$ are the average and standard deviation of the similarity within the pool, respectively. The parameter *α* was set to 1 for the high-rankers and to the ratio of the protein’s S score to that of the top ranker for the low-rankers. When calculating TM-score between 2 protein structures, only the residues aligned to the target sequence by HHalign were considered, and the target sequence length was used as the reference length. Finally, proteins dissimilar from the top ranker, with TM-score < 0.5, were removed [25], where the sequence length of the top ranker was used as the reference length for TM-score calculation.

### Multiple sequence alignment

Alignment between the target sequence and the template sequences was generated using PROMALS3D [18], one of the best multiple sequence alignment (MSA) tools available. PSI-BLAST [26] 2.1.14 was used with default parameters (number of iterations = 3, e-value cut = 0.001) for sequence profile generation. TM-align [20] and DaliLite [27] were used as structure-alignment tools to provide the 3D structure information required for PROMALS3D. Default values were used for all the other parameters of PROMALS3D. Less meaningful terminus regions were temporarily neglected in the initial MSA and attached afterwards. The less meaningful regions were defined as the termini of query sequence not aligned to any templates by the global alignment using HHsearch. We did not take those termini into consideration at this point because we assumed that they could be modeled reliably in the later *ab initio* refinement stage. By neglecting those regions, the alignment effort was focused on the more reliable core region, increasing the possibility of generating a more reliable model structure for the core.

### Model construction and optimization

Using the template structures and the MSA as input, a template-based model was constructed with the MODELLER-CSA [24] module, newly implemented in the GALAXY program package [11,14-16]. MODELLER-CSA is a template-based model-building procedure that carries out global optimization of the MODELLER restraint function [21] using conformational space annealing (CSA) [28-30]. In the new implementation in GALAXY, the MODELLER restraints are interpreted in the source code level and local minimization in the CSA procedure is performed by a quasi-Newton minimizer [31]. Both of these aspects are more advanced than the original implementation by Joo and coworkers [24]. A typical run of model building generated 100 structures that maximally satisfy the restraints. Among the 100 structures, the structure nearest to the largest cluster center was selected as a representative model.

### ULR detection and reconstruction

In the final stage, the single best template-based model structure was extensively refined by GalaxyREFINE [11], a high-resolution refinement method that employs advanced loop and terminus modeling algorithms [14,32,33]. Details of the refinement method can be found in Ref. 11, and here we describe it only briefly. ULRs were detected by a model-consensus quality assessment method [16]. The conformational space of ULR was then searched using a global optimization procedure that combines triaxial loop closure [32,33] and CSA on a newly introduced free energy surface composed of molecular mechanics force field [34], atomic-resolution statistical potential terms [35,36], and additional supporting terms. Information from templates was not used for scoring in the refinement procedure. All the energy components and the sampling algorithms were implemented in the GALAXY program.

In the current application of GalaxyREFINE to the model refinement in GalaxyTBM, a few modifications were made to enhance the computational efficiency over that of the original version used in CASP9. First, ULRs detected by the model consensus method [16] were subject to a filtering scheme that eliminates ULRs with less than 6 or more than 20 residues. Out of the remaining ULRs, up to 3 ULRs with lowest reliability (largest fluctuations among generated models) were subjected to actual reconstruction. Another change was that multiple ULRs were refined simultaneously in a single optimization procedure, while separate optimization for each ULR was performed and the results were merged into a single structure in CASP9. Finally, the initial loop structures for CSA were generated by a slightly different method from that used in CASP9. While all 30 starting loop structures were generated *de novo* by FALC [14] in CASP9, 5 loops were taken from initial template-based models and 25 loops were generated by FALC in the current implementation. This modification indirectly accounts for template information, which can be helpful when regions with reliable templates are assigned as ULRs.

### P-value calculation

P-values were obtained from paired two-tailed Student’s t-test.

## Competing interests

The authors declare that they have no competing interests.

## Authors’ contributions

All authors conceived of the study together. JK developed the template-based modelling method, wrote the scripts for the prediction pipeline, and drafted the manuscript. HB developed the refinement method and helped to draft the manuscript. CS participated in the design and coordination of the study and helped to draft the manuscript. All authors read and approved the final manuscript.
